# Advocating for data sharing in human genomics: an interview with Chris Wallace and Guillermo Reales

**DOI:** 10.1038/s42003-023-04947-3

**Published:** 2023-05-30

## Abstract

Dr. Chris Wallace is a Wellcome Trust Senior Research Fellow, Director of Research, and Principal Investigator in the Department of Medicine and a Programme Leader in the MRC Biostatistics Unit (BSU) at the University of Cambridge. Dr. Guillermo Reales is a postdoctoral researcher in Dr. Wallace’s group, where he recently co-authored a Comment evaluating the impact of sharing summary statistics on average citation rates of genome-wide association studies (GWASs). In this Q&A, we discussed the inspiration for their recent analysis on GWAS summary statistics, the importance of open data, and potential barriers or paths to data sharing in genomics.

**Figure:**
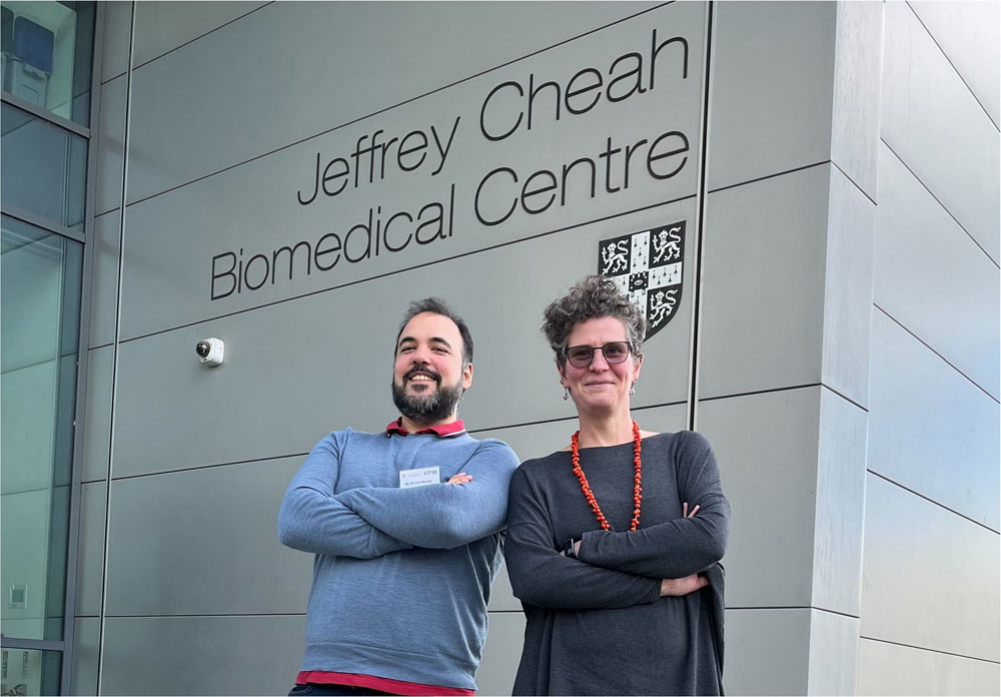
Guillermo Reales. Pictured from left to right: Guillermo Reales and Chris Wallace

Could you please tell us a little about yourselves?

**Chris Wallace (CW):** I was always interested in biology, how an organism “works”, but was directed to maths at school. I studied maths at undergraduate and took a Masters in statistics before working my way back to biology with a PhD thesis on the genetic susceptibility to leprosy (back in the days of linkage studies, and before anyone had heard of “GWAS”!). I became increasingly interested in the human immune system, and how maths and statistics could help understand the high-dimensional data becoming abundant in biology. I am lucky to now have a dry lab in two home departments - Cambridge Institute for Therapeutic Immunology & Infectious Disease (CITIID) which houses enormous expertise in immunology, and the MRC BSU which is one of the largest groups of biostatisticians in Europe. As a dry group, we use a variety of data science approaches to understand the shared and distinct factors underlying immune-mediated diseases.

**Guillermo Reales (GR):** I studied undergraduate biology in Spain, where I got interested in genetics. During my MSc, I trained in population genetics, when I worked on human populations from Spain and Latin America (including my first approximation to GWAS). Then I got the chance to move to Brazil for my PhD in Genetics, where I changed tracks and studied the molecular evolution of nervous system genes in neotropical primates. Along the way, I taught myself to program in R and grew more aware of the power of bioinformatics to make sense of the ever-growing amounts of genetic data generated, and to answer relevant biological questions. Then, after my PhD, I was still interested in humans and wanted to contribute to human health, so I joined Chris’ group as a Research Associate in 2019, where we’ve been using and developing computational and statistical methods to learn more about the genetic architecture of immune-mediated diseases from GWAS and related sources of data.

You recently published a Comment with us reporting that GWASs that share their summary statistics are (on average) cited more. What originally drove you to investigate this question (and was it really based on a Twitter thread)?

**GR:** Yes, that’s absolutely true! Twitter is (or was?) a fantastic tool for science if you get past all the not-so-nice things that come with it. One of my first realisations, when I started in the GWAS business, is how frustrating getting even summary data can be, and the lack of standardisation, which makes things harder even when you get your hands on the data (thanks a lot, GWAS Catalog, you’re a good friend!). Then Chris tweeted her frustrations, and I encouraged her to take the challenge – although we later realised she was maybe a bit optimistic!

**CW:** We make use of public data all the time, as well as data generated through collaborations with wet lab scientists. We understand the effort that goes into making large datasets, and are always grateful to the people who share data. We love to work in collaboration with the data generators where they are open to that, because they are the experts on their domain. But sometimes, you can be chasing datasets for a particular questions, and even though the papers are out there, they don’t address your question and you can’t find the data. I think on one of those afternoons I tweeted my frustration and Guillermo picked up on it, and ran with it. (Note, we do also communicate off Twitter!).

Your study of course makes the case that one benefit for data sharing is that it results in increased citations. What do you view as other benefits for authors who share their data?

**GR:** Unfortunately, we operate a system where scientists are evaluated by convenient yet imperfect and questionable metrics. In my view, sharing data is not so much about personal benefits as a scientist but rather about benefits for the whole community and ultimately for the patients and humanity in general. But, of course, this is very hard to measure, while the pressure to publish and be cited to progress in our careers is too tangible. I think it would be great if we could come up with new metrics to acknowledge the efforts of generating data and release them in a usable format for other people to do research on them.

**CW:** One benefit that I always stress to collaborators is that it removes the need to archive your own data. Data is precious, and you may want to go back to old datasets to build on them or answer new questions with them. But that can be difficult with time-limited funding and people moving institutions. If you are not careful, data gets lost, hard drives fail, or files are obscurely named and you’re not completely sure which was the final, post-QC version. Once a dataset is publicly archived (even behind a data access application), it is safe. You can retrieve the final, cleaned, documented version yourself as easily as anyone else can. It also feels genuinely positive when other people think your data can help answer their question, like you are contributing to a big team.

On the flip-side, what do you think are barriers to the deposition of summary statistics, even among authors who would like to share their datasets?

**GR:** It would be interesting to have more studies on this, but I think it’s a combination of several things. I think that in many cases, people are concerned about privacy, especially in large cohorts with public and private collaborations, where the decision of what data can and cannot be shared is complex. Then, preparing datasets for sharing and documenting them is tedious and often exhausting work, which usually comes at the end of the project, when people have many more interesting things to do. Lastly, I think we still lack a strong culture of open data sharing, where these sharing efforts are more widely considered an integral part of the work, rather than a complementary thing – we’re slowly getting there, though!

**CW:** Guillermo sums it up nicely. We shouldn’t underestimate the additional effort required to share data. Funders and institutions could help here, providing training in how to document and structure data and even support in reviewing and sharing datasets.

Beyond data sharing, are there any other topics that you think deserve more discussion and consideration among researchers in the field?

**GR:** I would say representativity is a key aspect we need to work on as scientists. An overwhelming majority of genetic studies have been realised in fully developed countries, comprising mostly European individuals. However, most of the world doesn’t have European ancestry, and differences in allele frequencies and linkage disequilibrium mean that, for example, genetic tools trained on Europeans don’t work well in other ancestries. Thus, we have an imbalanced knowledge landscape that we need to correct to ensure genetic advances serve everyone. Fortunately, there are some projects, like the African Genome Variation Project addressing this issue, but we still have a long way to go.

**CW:** Yes, it’s great to see new genetic initiatives being led now by scientists from Africa, such as the Ugandan Genome Resource, and Asia, such as Japan Biobank, but the years of investment overly-focused on populations of European-ancestry mean there is still a substantial gap. I also support open science more broadly, for example sharing not just data but the code used for its analysis. I think this helps with reproducibility, but also makes it easier to understand what analysis was done, when Methods can be rather brief. Again though, this can take additional time to do, and people may be nervous that their code doesn’t look perfect. We really need to change the culture so that people who share data, and people who share code, get credit for that extra work.

*This interview was conducted by Senior Editor, George Inglis*.

